# Vocal warm-up and breathing training for teachers: randomized clinical trial

**DOI:** 10.1590/S0034-8910.2015049005716

**Published:** 2015-09-23

**Authors:** Lílian Paternostro de Pina Pereira, Maria Lúcia Vaz Masson, Fernando Martins Carvalho

**Affiliations:** I Departamento de Fonoaudiologia. Universidade Federal da Bahia. Salvador, BA, Brasil; II Departamento de Medicina Preventiva e Social. Universidade Federal da Bahia. Salvador, BA, Brasil

**Keywords:** Faculty, Voice Quality, Hoarseness, prevention & control, Breathing Exercises, Occupational Health

## Abstract

**OBJECTIVE:**

To compare the effectiveness of two speech therapy interventions, vocal warm-up and breathing training, focusing on teachers’ voice quality.

**METHODS:**

A single-blind, randomized, parallel clinical trial was conducted. The research included 31 20 to 60-year old teachers from a public school in Salvador, BA, Northeasatern Brazil, with minimum workloads of 20 hours a week, who have or have not reported having vocal alterations. The exclusion criteria were the following: being a smoker, excessive alcohol consumption, receiving additional speech therapy assistance while taking part in the study, being affected by upper respiratory tract infections, professional use of the voice in another activity, neurological disorders, and history of cardiopulmonary pathologies. The subjects were distributed through simple randomization in groups vocal warm-up (n = 14) and breathing training (n = 17). The teachers’ voice quality was subjectively evaluated through the Voice Handicap Index (*Índice de Desvantagem Vocal*, in the Brazilian version) and computerized voice analysis (average fundamental frequency, jitter, shimmer, noise, and glottal-to-noise excitation ratio) by speech therapists.

**RESULTS:**

Before the interventions, the groups were similar regarding sociodemographic characteristics, teaching activities, and vocal quality. The variations before and after the intervention in self-assessment and acoustic voice indicators have not significantly differed between the groups. In the comparison between groups before and after the six-week interventions, significant reductions in the Voice Handicap Index of subjects in both groups were observed, as wells as reduced average fundamental frequencies in the vocal warm-up group and increased shimmer in the breathing training group. Subjects from the vocal warm-up group reported speaking more easily and having their voices more improved in a general way as compared to the breathing training group.

**CONCLUSIONS:**

Both interventions were similar regarding their effects on the teachers’ voice quality. However, each contribution has individually contributed to improve the teachers’ voice quality, especially the vocal warm-up.

## INTRODUCTION

Teachers use their voices as their main resource to transmit knowledge and emotion to their students, and they must keep a good vocal quality to ensure the communication efficiency that is inherent to the teaching-learning process.^16^

The combination between using the voice for long periods and occupational hazard factors (loud noises, improper ventilation, excessive working hours, lack of autonomy, lack of knowledge regarding proper vocal techniques, among others) contributes to make this one of the professional categories which is most frequently affected by voice disorders, which represents huge losses for teachers, school communities, and society.^8^ ^,^ ^a^

Observational epidemiological studies confirm the high prevalence of vocal disorders in teachers from various parts of the world, suggesting a multifactorial etiology. A study conducted in 27 Brazilian states found a higher average of vocal symptoms in teachers (3.7) as compared to other professionals (1.7); 63.1% of teachers mentioned having vocal alteration histories.^3^

Nevertheless, the number of controlled experimental studies aiming to investigate the effects from vocal interventions to produce evidence that contributes to raise awareness to, protect, and recover the vocal health of teachers.^6^

This study aimed to compare the effectiveness of two speech therapy interventions, vocal warm-up (VWU) and breathing training (BT), focusing on teachers’ voice quality.

## METHODS

A single-blind, randomized, parallel clinical trial was conducted. The data were collected from July to September 2013 in a large public school in Salvador, BA, Northeastern Brazil. The sample comprised 120 teachers and 2,300 middle and high school students.

Accidental sampling was used to choose the population, and all teachers in the school were invited to take part - they were selected according to certain eligibility criteria.

The inclusion criteria established ages between 20 and 60 years, minimum workloads of 20 hours a week, and either reporting or not reporting having vocal alterations.

The exclusion criteria were the following: being a smoker, excessive alcohol consumption, receiving additional speech therapy assistance while taking part in the study, being affected by upper respiratory tract infections, professional use of the voice in another activity, neurological disorders, and history of cardiopulmonary pathologies.

The teachers who reported not having fully complied with the proposed approach were excluded from the data analysis at the end of the interventions.

The monitored period lasted for six weeks (the same length that was used by other experiments).^15^ ^,^ ^18^ The team was previously trained to ensure consistency in their individual and collective procedures, and it has monitored the teachers daily throughout the whole intervention period.

First, the consenting teachers who met all eligibility criteria were informed about the study objective and the confidentiality of results. They were asked to fill out consent forms and sociodemographic questionnaires on their teaching activities and their vocal statuses, to characterize the sample.

The subjects were distributed through simple randomization in VWU and BT groups. Excel software (2007 version) was used to generate random numbers. The subjects’ data were then randomized and coded.

Out of 120 teachers invited, 41 of them (4.2%) stated being interested in taking part, met the eligibility criteria, and were randomized in VWU (n = 20) and BT (n = 21) groups. Five subjects withdrew from the study before the interventions, and two of them left during the monitored period; another three teachers were excluded from the data analysis for having reported not to have fully complied with the proposed approach. Thirty-one subjects that were randomized in VWU (n = 14) and BT (n = 17) groups remained through all research stages (Figure).

**Figure:**
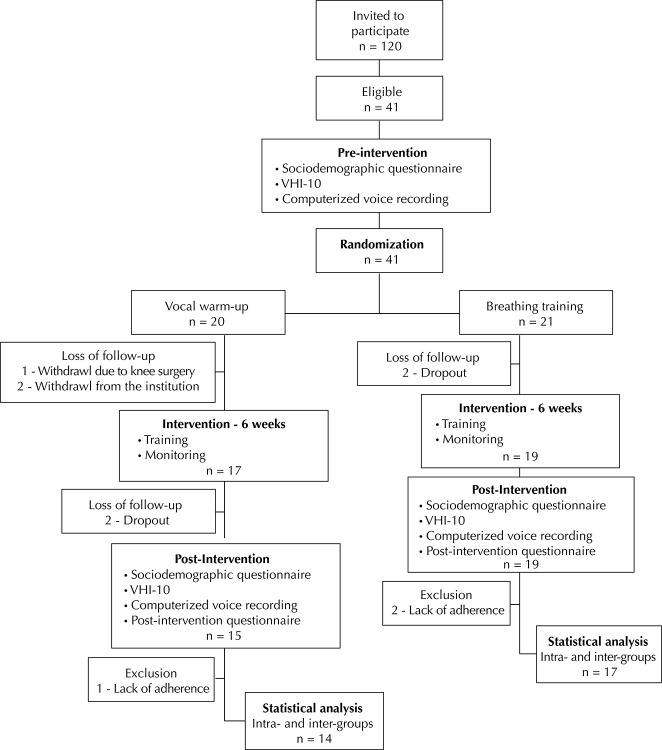
Flowchart for the selection and monitoring of research subjects. Salvador, BA, Northeastern Brazil, 2013.

Before the interventions, the groups did not significantly differ (p < 0.05) in regards to average ages, lengths of teaching experience, hours using their voices in teaching, genders, education levels, weekly workloads of 40 hours, self-reported vocal alterations, search for specialized treatment, fatigue whilst speaking, everyday water intake, and alcohol consumption (Table 1).

**Table 1:** Sociodemographic characterization of the teaching activity and the vocal conditions of teachers, before the intervention. Salvador, BA, Northeastern Brazil, 2013.

Variable	Vocal warm-up	Breathing training	p
	n = 14	n = 17	
Age in years (SD)	45.8 (SD = 8.1)	43.6 (SD = 11.4)	0.56a
Lengths of teaching experience (SD)	19.4 (SD = 8.5)	15.8 (SD = 7.8)	0.23^a^
Hours/day using the voice (SD)	8.4 (SD = 3.8)	8.0 (SD = 2.5)	0.71^a^
Gender (%)			
Female	85.8	70.6	0.41^b^
Male	14.2	29.4	
Education level (%)			
Undergraduate studies	7.1	35.3	0.09^b^
Graduate studies	92.9	64.7	
Workload (hours)			
< 40	21.4	11.8	0.88^b^
40	57.2	64.7	
> 40	21.4	23.5	
Self-reported vocal alteration (%)			
Yes	57.2	58.8	1.00^b^
No	42.8	41.2	
Sought specialized treatment for vocal alterations			
Yes	14.2	41.2	0.13^b^
No	85.8	58.8	
Fatigue whilst speaking (%)			
Yes	21.4	47.0	0.26^b^
No	78.6	53.0	
Everyday water intake (%)			
Yes	64.3	70.6	1.00^b^
No	35.7	29.4	
Alcohol consumption (%)			
Yes	35.7	47.0	0.72^b^
No	64.3	53.0	

^a^ Student t-test.

^b^ Fisher’s exact test.

The teachers’ voice quality was investigated through the subjects’ self-assessment and through computerized voice analysis by speech therapists.

To self-evaluate their voices, the subjects answered simplified, Brazilian Portuguese-validated Voice Handicap Index (VHI-10) questionnaires^5^ before and after the procedures were conducted. This questionnaire comprises a five-point Likert scale and is as reliable as the original version. It is frequently used in clinical trials.^15^ ^,^ ^18^ The score is calculated by simply adding up its points – the higher the score, the higher is the subject’s voice handicap. The averages of final VHI-10 scores were transformed in percentages to enable comparison with other studies and for voice handicap extents to be more easily visualized.

At the end of the monitored period, the subjects also filled out a post-intervention questionnaire,^15^ which evaluated interventions regarding treatment compliance, ease of speaking, confidence in the proposed approach, and clarity of speech.

The computerized voice analysis was performed individually, before and after the monitored period, by three assistant speech therapists (only at this study stage) who were blinded regarding the subjects’ intervention types.

The voice samples were recorded in VoxMetria software (version 4.7h from CTS Informática). It was installed in a Sony VAIO^®^ laptop that was equipped with an Intel^®^ Core™ i3 processor and a 64-bit Realtek sound card with a recording speed of 20 KHz. The recording was conducted in a room outside the main school area, in a properly calibrated OTOBEL, BEL-BABY2 audiometric booth that was certified by INMETRO (Brazilian National Institute of Metrology, Quality and Technology).

A SHURE, model WH20 dynamic cardioid headset was used, and it was placed 4 cm away from the speaker’s mouth at a 45° angle, according to the software user’s manual. The teachers were asked to remain sitting and emit sustained vowel /Ɛ:/ in the same pitch and intensity they were used to, for five seconds.

The values of the following voice parameters were extracted from the software after the initial and final segments of tracks were excluded (due to their inherent instability): average fundamental frequency (F_0_); short-term disturbance measurements – jitter (variance in the fundamental frequency at each cycle) and shimmer (wavelength variance at each cycle); noise and glottal-to-noise excitation ratio (GNE).

The normal voice limits were indicated by VoxMetria acoustic analysis software: jitter (< 0.6%); shimmer (< 6.5%); noise (< 2.5 dB), and GNE ratio (≥ 0,5 dB).

All the subjects were told not to perform the exercise sequence on the day of the final acoustic recording, as the analysis intended to verify the effects of long-term interventions.

The initial training and the monitoring of teachers were conducted by three experienced speech therapists in both intervention approaches. These therapists only took part in the study stage.

At the end of the monitored period, each subject received a report on their improvement and regarding their need to look for medical assistance.

The intervention that was conducted in the VWU group lasted 13 minutes in average, and the subjects were instructed to perform its exercises once a day, before they would teach their first classes, in order that they maintain the immediate vocal effects to achieve higher efficiency while working.^12^ The exercise program included: stretching of upper limbs (rotating shoulders, raising arms, and stretching the neck); long utterance of fricative phonemes /z:/ and /s:/ to direct the airflow; vibrant sounds at usual pitch, with modulation of frequencies (vibrating tongue or lips); and nasal sounds for higher vocal projection and resonance anteriorization.^12^

The intervention in the BT group was adapted from a previous study, and it involved the performance of exercises for the muscles of exhalation through a mucus clearance device (New Shaker, NCS brand) coupled with a nasal clip, lasting 13 minutes in average.^15^ The exercises included breathing through the mouth with nostrils closed by the clip, and deep exhaling through the mucus clearance device. Five series with five repetitions each were performed, with 15 to 30-second pauses between each of them. Each subject was instructed to perform the exercise sequence once a day, before they taught their first classes. All subjects in that group received a mucus clearance device.

The self-assessment (VHI-10) and the computerized voice analysis were considered as primary outcomes. Treatment adherence and intervention benefits, which were obtained from the post-intervention questionnaire, were considered as secondary outcomes. The analyses (before and after the interventions) were conducted by comparing results within groups and from one group to the other.

The statistical analyses were conducted in a blinded way. Statistical Package for the Social Sciences (SPSS, version 19.0) was used to store the data and conduct hypothesis testing. A 5% significance level was adopted (p ≤ 0.05). Student t-test for independent samples and Fisher’s exact test were used to compare the characteristics of the groups before the intervention. Wilcoxon signed-rank test was used to compare the values of self-assessment and acoustic voice indicators before and after the intervention. Mann–Whitney U test was used to compare the differences in self-assessment and acoustic voice indicators between VWU and BT groups before and after the intervention.

The study was conducted pursuant to the Declaration of Helsinki, and it was approved by the Research Ethics Committee of Faculdade de Medicina da Bahia (Process 234,154, from April 1, 2013). It was registered on ClinicalTrials.gov under the identifying number NCT02102399.

## RESULTS

Comparisons within the VWU group, before and after the intervention, showed a statistically significant reduction of self-assessed (VHI-10) and acoustic vocal indicators (F_0_ average), which was not observed in the other analyzed parameters, nonetheless (Table 2).

**Table 2:** Self-assessed and acoustic voice indicators of the teachers from a public school, according to the groups and intervention periods. Salvador, BA, Northeastern Brazil, 2013.

Indicator	Vocal warm-up					Breathing training				
	Before		After		p	Before		After		p
	Average	SD	Average	SD		Average	SD	Average	SD	
VHI (%)	21.78	17.44	13.04	14.18	0.007*	20.44	13.70	13.82	10.86	0.001*
F_0_ (Hz)	196.21	34.47	186.25	31.53	0.049*	191.87	43.35	185.71	33.70	0.345
Jitter (%)	0.14	0.11	0.10	0.05	0.323	0.19	0.14	0.45	0.94	0.171
Shimmer (%)	2.90	0.85	3.10	0.97	0.296	2.77	0.94	4.19	2.60	0.022*
Noise (dB)	0.69	0.50	0.68	0.50	0.776	0.72	0.46	0.72	0.44	0.451
GNE (dB)	0.89	0.12	0.92	0.06	0.959	0.88	0.11	0.88	0.11	0.477

VHI: Voice Handicap Index (Índice de Desvantagem Vocal); F_0_: Fundamental frequency; GNE: Glottal to Noise Excitation ratio

* Wilcoxon’s signed-rank test.

The comparisons within the BT group, before and after the intervention, found a statistically significant reduction (p < 0.001) in the self-assessed vocal indicator (VHI-10) and in the shimmer acoustic indicator (p = 0.002). The remaining parameters investigated were not found to differ significantly (p < 0.05) (Table 2).

The averages of the acoustic analysis parameters from the groups (jitter, shimmer, noise, and GNE ratio) were found to be placed within normal parameters before and after the interventions (Table 2).

The variation in self-assessed and acoustic voice indicators was similar between VWU and BT groups, when they were compared at times before and after the intervention (Table 3).

**Table 3:** Average difference in values before and after the intervention for self-assessed and acoustic voice indicators of the teachers from a public school, according to the intervention groups. Salvador, BA, Northeastern Brazil, 2013.

Indicator	Vocal warm-up (n = 14)		Breathing training (n = 17)		
	Before		After		p*
	Average difference	SD	Average difference	SD	
VHI (%)	-8.75	10.13	-6.61	6.84	0.76
F_0_ (Hz)	-9.96	17.14	-6.15	26.11	0.30
Jitter (%)	-0.02	0.10	0.25	0.89	0.20
Shimmer (%)	0.20	0.89	1.41	2.30	0.77
Noise (dB)	-0.01	0.14	-0.004	0.67	0.49
GNE ratio (dB)	0.02	0.13	0.001	0.67	0.74

* Mann-Whitney U test.

General voice improvement (p = 0.003) and increased ease of speech (p = 0.030) were more frequently reported by subjects from the VWU group than by the ones in BT group, after the intervention (Table 4). Other benefits from the intervention (higher speech clarity and confidence in the intervention) were also more frequently reported by subjects in the VWU group. However, those differences were not statistically significant (p = 0.120 ad p = 0.107, respectively).

**Table 4:** Comparison among self-reported indicators on the intervention benefits within the groups for 31 public school teachers. Salvador, BA, Northeastern Brazil, 2013.

Indicator	Vocal warm-up		Breathing training		p
	n = 14	%	n = 17	%	
General voice improvement					
Yes	9	64.3	2	11.8	0.003*
No	5	35.7	15	88.2	
Improved speech clarity					
Yes	7	50.0	3	17.7	0.120
No	7	50.0	14	82.3	
Higher ease of speech					
Yes	8	57.2	3	17.7	0.030*
No	6	42.8	14	82.3	
Confidence in the intervention					
Yes	14	100	13	76.5	0.107
No	0	0	4	23.5	

* Fisher’s exact test.

Good or moderate self-reported adherence at the end of interventions was statistically similar (p = 0.269) in VWU group (71.5%) and in BT group (82.3%). 28.6% of subjects in the VWU group and 17.6% of subjects in the BT group reported having had low adherence to treatments.

## DISCUSSION

The studied speech therapy interventions, vocal warm-up and breathing training, similarly improved the teachers’ voice quality.

The average VHI-10 scores which were observed for the groups before the intervention were considered to be within the percentage voice range that is seen as the proper one for subjects with vocal symptoms,^5^ in spite of the existence of some high standard deviation values.

The reduction in VHI-10 scores that was observed in the analyses within groups was similar to the one from a study^18^ that also confirmed their reduction for both the direct vocal training and in the breathing training one, even though different professional categories have taken part in the interventions.

The Hawthorne^9^ effect might have contributed to the significant reduction in the voice handicap indices within the intervention groups, when one considers the attention and concern with the vocal health of teachers, according to the research objectives and procedures. The workers may have felt they were being valued and led to a positive behavioral change.

The VHI-10 scores did not differ significantly according to the intervention types, VWU or BT (Table 3). It was not possible to find any of the interventions to be better than the other one regarding the self-reported vocal parameters that were dealt with in that protocol.

The statistically significant reduction in the average of F_0_ that was observed in the VWU group suggests a probable decreased vocal effort and overworked larynx muscles in the subjects of this group, and that was a positive effect from the increased protection to the vocal health in the teaching routine. Some studies correlate the reduction in F_0_ with the decreased voice strain and the more relaxed speech after the vocal exercises are performed.^13^ ^,^ ^14^ ^,^ ^17^

The results from this study indicate a possible protecting effect from vocal warm-up, as the increase in F_0_ could be inversely related to the increased number of vibration cycles in the vocal folds, which triggers augmented vocal friction and fatigue, larynx discomfort, and vocal effort after the vocal overload activity.^14^ ^,^ ^17^

The reduction in F_0_ has also been already confirmed^10^ after posture and cervical relaxation exercises were performed by teachers who were given training, as compared to the control group, which was given an indirect approach, which confirms the voice strain reduction. The VWU program included posture exercises, neck and shoulder stretching exercises, which may have contributed to the reduction in F_0_.

The opposite of what was expected was found in the analysis within the BT group. The subjects in this group were observed to have their cycle-to-cycle vibration extent variance and disturbance average measurements (shimmer) significantly increased, which is contrary to the expectation that the breathing training would have provided increased breathing control at exhalation, as increased breathing exhalation muscle conditioning can be inferred. That indicates increased voice instability.^2^

The computerized voice acoustic analysis, at moments before and after the intervention, have not differed significantly among VWU and BT groups.

The VWU was expected to have a higher effectiveness in vocal quality, which indicates its superiority as compared to BT. The number of subjects might not have been enough, which may have induced a type II error (false negative).

Most teachers in the VWU group reported having their voices improved and increased ease of speech after the monitored period, and a statistically significant difference was found as compared to the BT group. Those results corroborate other experiments^4^ ^,^ ^15^ that found greater benefits (general voice improvement and improved dysphonia) as reported by teachers who were submitted to a direct intervention approach.

The self-reported benefits from VWU group may be related to the direct recruitment of speech muscles, which was promoted by vibrational, fricative, and resonance exercises aiming to balance emission and provide higher projection, resistance, and flexibility for the use of voice for extended lengths of time.^7^ ^,^ ^10^

On the other hand, the fact that BT plays an indirect role in the speech organs, i.e., through the recruitment of muscles of exhalation,^5^ ^,^ ^18^ may have made, to a certain extent, the subjects in this group fail to observe benefits in their speech function.

The sampling in the study was not probabilistic or accidental, which hindered generalizing the results for the teacher population. However, the randomization implies the assurance that all confounding variables have equal chances to be allocated in any of the groups, which thus makes them comparable and minimizes selection and confounding biases.

The vocal analysis does not have a single outcome that is strong enough to indicate voice changes, but several outcomes are used, and those may induce to the type I error (false positive).

In the last week of the monitored period, the teachers were found to have lesser vocal demands due to the absence of cleaning staff at the school. However, it should be said that the subjects from both randomized groups were under the same condition.

The healthy worker effect, which is common to occupational studies, may justify the low number of teachers with moderate or intense self-reported vocal alterations before the interventions. Those subjects might have been taking leaves of absence, they might have looked for other occupations, or they might have abandoned their profession due to their speech symptoms. The real prevalence of the studied phenomenon may have been underestimated.^1^ ^,^ ^16^

The subjects in the VWU group may have reported higher benefits from the vocal warm-up in the post-intervention questionnaire due to being psychologically influenced by the increased expectation for improved vocal quality with this speech therapy approach. However, the acoustic analysis of objective voice parameters overcomes this probable bias.

Even though they were a minority, the teachers who reported low adherence to the proposed approach may have negatively contributed to the comparative analysis of intervention effects. Adherence to voice therapies is known to be a challenge, and it depends on expected results, interest, and confidence in improving vocal behavior; it may interfere in the treatment prognosis and evolution.^11^

The intervention groups are concluded not to differ significantly regarding self-assessed and acoustic voice indicators. It was not possible to point out one of the approaches as being the most effective one or offering improved protection to the vocal health of teachers. However, both vocal warm-up and breathing training individually caused VHI-10 to reduce, as reported by the teachers. The subjects in the VWU group were found to have reduced F_0_ and they reported having increased benefits from the intervention regarding the general improvement of their voices and ease of speech after the monitored period.

To confirm our results, the conduction of other experimental studies on the topic, with more subjects and involving teachers from different schools, is recommended.

## Footnotes

a Ministério da Saúde, Secretaria de Vigilância em Saúde, Departamento de Saúde Ambiental e Saúde do Trabalhador. Distúrbio de voz relacionado ao trabalho. Brasília (DF); 2011 [cited 2015 Apr 26]. (Série A. Normas e Manuais Técnicos). Available from: http://aborlccf.org.br/imagebank/Protocolo_DVRT.pdf
