# Supplementary material for: Vocal warm-up and breathing training for teachers: randomized clinical trial
Source: Rev Saude Publica. 2015 Sep 23;49:67. doi: 10.1590/S0034-8910.2015049005716 (PMC4587821; doi:10.1590/S0034-8910.2015049005716)
Supplement: Translation

# Translation: Aquecimento vocal e treino respiratório em professores: ensaio clínico randomizado

**Authors:** Lílian Paternostro de Pina Pereira, Maria Lúcia Vaz Masson, Fernando Martins Carvalho

**Affiliations:** I Departamento de Fonoaudiologia. Universidade Federal da Bahia. Salvador, BA, Brasil; II Departamento de Medicina Preventiva e Social. Universidade Federal da Bahia. Salvador, BA, Brasil

**Keywords:** Docentes, Qualidade da Voz, Rouquidão, prevenção & controle, Exercícios Respiratórios, Saúde do Trabalhador

## Abstract

**OBJETIVO:**

Comparar a efetividade de duas intervenções fonoaudiológicas, aquecimento vocal e treino respiratório, na qualidade vocal de professores.

**MÉTODOS:**

Realizou-se ensaio clínico randomizado, cego ao avaliador, com delineamento em paralelo 1:1. Participaram da pesquisa 31 professores da rede estadual de ensino de Salvador, BA, entre 20 e 60 anos de idade, carga laboral mínima de 20h semanais, com ou sem alteração vocal autorreferida. Os critérios de exclusão foram: tabagismo, elevada frequência de consumo de álcool, acompanhamento fonoaudiológico simultâneo à participação no estudo, estado de infecções de trato respiratório superior, uso profissional da voz em outra atividade, distúrbios neurológicos e histórico de patologias cardiopulmonares. Os indivíduos foram alocados por randomização simples nos grupos aquecimento vocal (n = 14) e treino respiratório (n = 17). A qualidade vocal dos professores foi avaliada subjetivamente pelo Índice de Desvantagem Vocal e pela análise acústica computadorizada da voz (frequência fundamental média, *jitter*, *shimmer*, ruído e proporção *glottal-to-noise excitation*), realizada por fonoaudiólogos.

**RESULTADOS:**

Antes das intervenções, os grupos eram semelhantes quanto às características sociodemográficas, atividade docente e qualidade vocal. As variações pré e pós-intervenção nos indicadores de autoavaliação e acústicos da voz não diferiram significantemente entre os grupos. Na comparação intragrupos, pré e pós-intervenções de seis semanas, observou-se redução significativa do Índice de Desvantagem Vocal nos participantes dos dois grupos, redução da frequência fundamental média no grupo aquecimento vocal e aumento do *shimmer* no grupo treino respiratório. Indivíduos do grupo aquecimento vocal referiram maior facilidade para falar e maior benefício em relação à melhoria geral da voz, comparado ao grupo treino respiratório.

**CONCLUSÕES:**

Ambas intervenções foram similares quanto a seus efeitos sobre a qualidade vocal dos professores. Entretanto, cada intervenção isoladamente, em especial o aquecimento vocal, contribuiu para melhorar a qualidade vocal dos professores.

## INTRODUÇÃO

Professores utilizam a voz como principal recurso para transmitir conhecimento e emoção aos alunos, devendo manter boa qualidade vocal para garantir a eficiência da comunicação inerente ao adequado processo de ensino-aprendizagem.^16^

A combinação do uso prolongado da voz e fatores de risco ocupacionais (ruído elevado, ventilação inadequada, jornada excessiva, falta de autonomia, desconhecimento de técnicas vocais adequadas, dentre outros) contribui para que essa categoria profissional seja uma das mais acometidas por problemas de voz, representando enormes prejuízos para o docente, a comunidade escolar e a sociedade.^8^ ^,^ ^a^

Pesquisas epidemiológicas observacionais ratificam a elevada prevalência de problemas vocais em professores de diversas partes do mundo e sugerem etiologia multifatorial. Estudo desenvolvido nos 27 estados brasileiros constatou média maior de sintomas vocais em professores (3,7) em relação aos não professores (1,7) e 63,1% dos docentes referiram histórico de alteração vocal.^3^

No entanto, ainda é incipiente o número de estudos experimentais controlados que têm o objetivo de investigar os efeitos de intervenções vocais para a obtenção de evidências que contribuam para ações de promoção, proteção e recuperação da saúde vocal de docentes.^6^

Este estudo objetivou comparar a efetividade de duas intervenções fonoaudiológicas, aquecimento vocal (AV) e treino respiratório (TR), na qualidade vocal de professores.

## MÉTODOS

Realizou-se ensaio clínico randomizado, cego ao avaliador, com delineamento em paralelo 1:1. A coleta de dados ocorreu de julho a setembro de 2013 em uma escola de grande porte da rede estadual de ensino de Salvador, BA, composta por 120 professores e 2.300 alunos dos níveis Fundamental II e Ensino Médio.

A amostra populacional foi escolhida por critério de conveniência, sendo todos os professores da escola convidados a participar e selecionados de acordo com critérios de elegibilidade.

Como critérios de inclusão, admitiu-se idade entre 20 e 60 anos, carga laboral mínima de 20h semanais, com ou sem alteração vocal autorreferida.

Os critérios de exclusão foram: tabagismo, elevada frequência de consumo de álcool, acompanhamento fonoaudiológico simultâneo à participação no estudo, estado de infecções de trato respiratório superior, uso profissional da voz em outra atividade, distúrbios neurológicos e histórico de patologias cardiopulmonares.

Os professores que relataram ausência total de adesão à abordagem proposta, ao final das intervenções, foram excluídos da análise dos dados.

O período de seguimento foi de seis semanas, tempo utilizado por outras pesquisas experimentais.^15^ ^,^ ^18^ A equipe recebeu capacitação prévia para garantir a homogeneidade dos procedimentos intra e interpesquisadores e monitorou os professores diariamente, durante todo o período de intervenção.

No primeiro momento, os professores concordantes e que satisfizeram os critérios de elegibilidade foram esclarecidos sobre o objetivo do estudo, confidencialidade dos resultados e solicitados a preencher o termo de consentimento livre e esclarecido e questionário sociodemográfico, da atividade docente e da condição vocal, para a caracterização da amostra.

Os participantes foram alocados por randomização simples nos grupos AV e TR, tendo sido utilizado o programa Excel (versão 2007) para gerar números aleatórios. Foi realizada a randomização e a codificação dos dados dos participantes.

Do total de 120 professores convidados, 41 (4,2%) manifestaram interesse em participar, atendiam aos critérios de elegibilidade, e foram randomizados nos grupos AV (n = 20) e TR (n = 21). Ocorreram perdas de cinco sujeitos antes das intervenções e de dois durante o seguimento; mais três exclusões foram realizadas antes da análise dos dados devido ao relato de ausência total de adesão à abordagem proposta. Permaneceram 31 participantes, randomizados nos grupos AV (n = 14) e TR (n = 17) e que concluíram todas as etapas da pesquisa (Figura).

**Figura:** Fluxograma de seleção e acompanhamento dos participantes da pesquisa. Salvador, BA, 2013.

Antes das intervenções, os dois grupos não diferiram significantemente (p > 0,05) quanto à média de idade, tempo de docência, horas de uso profissional da voz, sexo, escolaridade, carga laboral de 40h semanais, alteração vocal autorreferida, busca por tratamento especializado, cansaço ao falar, ingestão habitual de água e uso de álcool (Tabela 1).

**Tabela 1:** Caracterização sociodemográfica da atividade docente e da condição vocal de professores, no momento pré-intervenção. Salvador, BA, 2013.

Variável	Aquecimento vocal	Treino respiratório	p
	n = 14	n = 17	
Idade em anos (DP)	45,8 (DP = 8,1)	43,6 (DP = 11,4)	0,56a
Tempo de docência em anos (DP)	19,4 (DP = 8,5)	15,8 (DP = 7,8)	0,23^a^
Horas/dia de uso da voz (DP)	8,4 (DP = 3,8)	8,0 (DP = 2,5)	0,71^a^
Sexo (%)			
Feminino	85,8	70,6	0,41^b^
Masculino	14,2	29,4	
Escolaridade (%)			
Graduação	7,1	35,3	0,09^b^
Pós-graduação	92,9	64,7	
Carga laboral (horas)			
< 40	21,4	11,8	0,88^b^
40	57,2	64,7	
> 40	21,4	23,5	
Alteração vocal autorreferida (%)			
Sim	57,2	58,8	1,00^b^
Não	42,8	41,2	
Buscou tratamento especializado para alteração vocal			
Sim	14,2	41,2	0,13^b^
Não	85,8	58,8	
Cansaço ao falar (%)			
Sim	21,4	47,0	0,26^b^
Não	78,6	53,0	
Ingestão habitual de água (%)			
Sim	64,3	70,6	1,00^b^
Não	35,7	29,4	
Uso de álcool (%)			
Sim	35,7	47,0	0,72^b^
Não	64,3	53,0	

^a^ Teste *t* de Student.

^b^ Teste Exato de Fisher.

A qualidade vocal dos professores foi investigada por autoavaliação dos sujeitos e análise acústica computadorizada, realizada por fonoaudiólogos.

Para a autoavaliação da voz, os participantes responderam ao protocolo Índice de Desvantagem Vocal (IDV-10) em seu formato simplificado, validado para o português,^5^ antes e após a realização dos procedimentos. Este protocolo é composto por uma escala *Likert* de cinco pontos e tem confiabilidade em relação à versão original, frequentemente utilizada em ensaios clínicos.^15^ ^,^ ^18^ O escore é calculado por soma simples e quanto maior o valor, maior a desvantagem vocal. As médias dos escores finais do IDV-10 foram transformadas em porcentagem para maior comparabilidade com outras pesquisas e fácil visualização da magnitude da desvantagem vocal.

Ao final do seguimento, os participantes também preencheram o questionário pós-intervenção^15^ que avaliou as intervenções quanto à adesão, melhora geral da voz, facilidade para falar, credibilidade na abordagem proposta e clareza da voz.

A análise acústica computadorizada da voz foi realizada individualmente, antes e após o seguimento, por três fonoaudiólogos auxiliares apenas nesta etapa do estudo e que desconheciam o tipo de intervenção dos participantes.

As amostras de voz foram gravadas no programa *VoxMetria* versão 4.7h da CTS Informática, instalado em *notebook* da marca SONY, modelo VAIO^®^, processador Intel^®^ Core™ i3, placa de som de 64 bits, da marca *Realtek* e com velocidade de gravação de 20 KHz. A gravação foi realizada em sala externa à área principal da escola e em cabina audiométrica compacta da marca OTOBEL, modelo BEL-BABY2, devidamente calibrada, com certificação pelo INMETRO.

O tipo de microfone utilizado foi *headset* cardioide dinâmico, marca SHURE, modelo WH20, posicionado a 4 cm da boca do falante e em ângulo de 45º, conforme orientação estabelecida no manual do programa. Os professores foram solicitados a permanecer em postura sentada e emitir a vogal sustentada /Ɛ:/ em tom e intensidade habitual por cinco segundos.

Os valores dos parâmetros acústicos da voz: frequência fundamental média (F_0_); medidas de perturbação a curto prazo – *jitter *(variabilidade da frequência fundamental ciclo a ciclo) e *shimmer *(variabilidade da amplitude ciclo a ciclo); ruído e proporção *glottal to noise excitation* (GNE) foram extraídos do programa após a exclusão dos trechos iniciais e finais das emissões devido à inerente instabilidade.

Os limites considerados dentro dos padrões de normalidade da voz foram os indicados pela normatização do programa de análise acústica (*VoxMetria*): *jitter *(< 0,6%)*; shimmer* (< 6,5%); ruído (< 2,5 dB) e proporção GNE (≥ 0,5 dB).

Todos os sujeitos foram orientados a não realizar a sequência de exercícios no dia da gravação acústica final, pois o intuito da análise foi a verificação dos efeitos das intervenções a longo prazo.

O treinamento inicial e monitoramento dos professores foram realizados por três fonoaudiólogos experientes nas duas abordagens de intervenção e que participaram apenas desta etapa do estudo.

Ao final do seguimento, cada participante recebeu relatório sobre os benefícios atingidos e possíveis encaminhamentos.

A intervenção realizada no grupo AV teve duração média de 13 min e os sujeitos foram orientados a realizá-la uma vez ao dia, antes de ministrar a primeira aula, no intuito de manutenção dos efeitos vocais imediatos para maior eficiência vocal no exercício docente.^12^ O programa de exercícios incluiu: alongamento e flexibilidade de membros superiores (rotação de ombros, elevação dos braços e alongamento cervical); expiração longa de fonemas fricativos /z:/ e /s:/ para direcionamento do fluxo aéreo; sons vibrantes em tom habitual e com modulação de frequência (vibração de língua ou lábios) e sons nasais para maior projeção vocal e anteriorização ressonantal.^12^

A intervenção no grupo TR foi adaptada de estudo anterior e envolveu a execução de exercícios da musculatura expiratória por meio de equipamento incentivador respiratório (marca NCS e modelo *New Shaker*), associado a clipe nasal, com duração média de 13 min.^15^ Os exercícios incluíram inspirações por via oral, com oclusão nasal por intermédio do clipe e expirações profundas via incentivador respiratório. Foram realizadas cinco séries de cinco repetições, com pausa de 15 a 30 segundos entre cada uma. Cada participante foi orientado a realizar a sequência de exercícios uma vez ao dia, antes de ministrar a primeira aula. Todos os participantes desse grupo receberam o equipamento incentivador respiratório.

A autoavaliação (IDV-10) e a análise acústica computadorizada da voz foram consideradas desfechos primários. Adesão e benefícios da intervenção, obtidos no questionário pós-intervenção, foram considerados desfechos secundários. As análises foram realizadas intra (pré e pós-intervenções) e intergrupos.

A análise estatística foi realizada de forma cega. Utilizou-se o programa *Statistical Package for the Social Sciences* (SPSS – versão 19.0) para o armazenamento dos dados e realização dos testes de hipótese, sendo adotado o grau de significância de 5% (p ≤ 0,05). O Teste t de Student para amostras independentes e o teste exato de Fisher foram utilizados para comparar características dos grupos, pré-intervenção. O *signed-rank test* de Wilcoxon foi utilizado para comparar valores dos indicadores de autoavaliação e acústicos da voz, pré e pós-intervenção. O teste de Mann-Whitney foi utilizado para comparar a diferença nos indicadores de autoavaliação e acústicos da voz, pré e pós-intervenção, entre os grupos AV e TR.

O estudo foi realizado de acordo com a Declaração de Helsinque e aprovado pelo Comitê de Ética em Pesquisa da Faculdade de Medicina da Bahia (Processo 234.154, de 1 de abril de 2013) e registrado no ClinicalTrials.gov sob identificador NCT02102399.

## RESULTADOS

A comparação pré e pós-intervenção intragrupo de AV mostrou redução estatisticamente significante dos indicadores vocais de autoavaliação (IDV-10) e acústico (F_0_ média), mas não nos outros parâmetros analisados (Tabela 2).

**Tabela 2:** Indicadores de autoavaliação e acústicos da voz de professores de escola pública, segundo grupo e período de intervenção. Salvador, BA, 2013.

Indicador	Aquecimento vocal					Treino respiratório				
	Pré		Pós		p	Pré		Pós		p
	Média	DP	Média	DP		Média	DP	Média	DP	
IDV (%)	21,78	17,44	13,04	14,18	0,007*	20,44	13,70	13,82	10,86	0,001*
F_0_ (Hz)	196,21	34,47	186,25	31,53	0,049*	191,87	43,35	185,71	33,70	0,345
*Jitter* (%)	0,14	0,11	0,10	0,05	0,323	0,19	0,14	0,45	0,94	0,171
*Shimmer* (%)	2,90	0,85	3,10	0,97	0,296	2,77	0,94	4,19	2,60	0,022*
Ruído (dB)	0,69	0,50	0,68	0,50	0,776	0,72	0,46	0,72	0,44	0,451
GNE (dB)	0,89	0,12	0,92	0,06	0,959	0,88	0,11	0,88	0,11	0,477

IDV: Índice de Desvantagem Vocal; F_0_: Frequência fundamental; GNE: *Glottal to Noise Excitation ratio*

* *Wilcoxon’s signed-rank test.*

Na comparação pré e pós-intervenção intragrupo de TR, constatou-se redução estatisticamente significante (p < 0,001) do indicador de autoavaliação vocal (IDV-10), e aumento do indicador acústico *shimmer* (p = 0,022). Os demais parâmetros investigados não diferiram significantemente (p > 0,05) (Tabela 2).

As médias dos parâmetros de análise acústica dos grupos (*jitter, shimmer*, ruído e proporção GNE) apresentaram-se dentro dos padrões de normalidade antes e após as intervenções (Tabela 2).

A variação nos indicadores de autoavaliação e acústicos da voz foi similar (p > 0,05) entre os grupos AV e TR, quando comparados nos momentos pré e pós-intervenção (Tabela 3).

**Tabela 3:** Diferença média nos valores pré e pós-intervenção dos indicadores de autoavaliação e acústicos da voz de professores de escola estadual, segundo grupo de intervenção. Salvador, BA, 2013.

Indicador	Aquecimento vocal (n = 14)		Treino respiratório (n = 17)		
	Pré		Pós		p*
	Diferença média	DP	Diferença média	DP	
IDV (%)	-8,75	10,13	-6,61	6,84	0,76
F_0_ (Hz)	-9,96	17,14	-6,15	26,11	0,30
*Jitter* (%)	-0,02	0,10	0,25	0,89	0,20
*Shimmer* (%)	0,20	0,89	1,41	2,30	0,77
Ruído (dB)	-0,01	0,14	-0,004	0,67	0,49
Proporção GNE (dB)	0,02	0,13	0,001	0,67	0,74

* Teste de Mann-Whitney.

Melhora geral da voz (p = 0,003) e maior facilidade para falar (p = 0,030) após a intervenção foram mais frequentemente relatados por indivíduos do grupo AV do que nos do grupo TR (Tabela 4). Outros benefícios da intervenção (maior clareza na voz e credibilidade na intervenção) também foram mais frequentemente relatados no grupo AV, porém essas diferenças não foram estatisticamente significantes (p = 0,120 e p = 0,107, respectivamente).

**Tabela 4:** Comparação entre indicadores de autoavaliação sobre os benefícios da intervenção; intergrupos em 31 professores de escola estadual. Salvador, BA, 2013.

Indicador	Aquecimento vocal		Treino respiratório		p
	n = 14	%	n = 17	%	
Melhora geral da voz					
Sim	9	64,3	2	11,8	0,003*
Não	5	35,7	15	88,2	
Maior clareza na voz					
Sim	7	50,0	3	17,7	0,120
Não	7	50,0	14	82,3	
Maior facilidade para falar					
Sim	8	57,2	3	17,7	0,030*
Não	6	42,8	14	82,3	
Credibilidade na intervenção					
Sim	14	100	13	76,5	0,107
Não	0	0	4	23,5	

* Teste Exato de Fisher.

Adesão boa ou moderada relatada ao final das intervenções foi estatisticamente semelhante (p = 0,269) no grupo AV (71,5%) e no grupo TR (82,3%). Afirmaram baixa adesão, 28,6% do grupo AV e 17,6% do grupo TR.

## DISCUSSÃO

As intervenções fonoaudiológicas pesquisadas, aquecimento vocal e treino respiratório, melhoraram, de forma similar, a qualidade vocal dos professores.

Os escores médios do IDV-10 apresentados pelos grupos antes da intervenção foram considerados dentro da faixa percentual de voz considerada adequada para indivíduos com queixas vocais,^5^ apesar da ocorrência de alguns valores elevados de desvios-padrão.

A redução dos escores do IDV-10 observada nas análises intragrupos foi similar a estudo^18^ que também confirmou sua redução tanto no grupo de terapia vocal direta, como naquele de treino respiratório, embora diferentes categorias de profissionais da voz tenham participado das intervenções.

O efeito Hawthorne^9^ pode ter contribuído para a redução significativa dos índices de desvantagem vocal intragrupos de intervenção, considerando-se a atenção e preocupação com a saúde vocal dos professores, segundo os objetivos e procedimentos da pesquisa. Os trabalhadores podem ter-se sentido valorizados, sendo levados à mudança positiva de comportamento.

Os escores do IDV-10 não diferiram significantemente segundo o tipo de intervenção AV ou TR (Tabela 3), não sendo possível constatar a superioridade de uma das intervenções em relação aos parâmetros vocais autorreferidos, abordados nesse protocolo.

A redução estatisticamente significante da média da F_0_ ocorrida intragrupo de AV sugere provável diminuição do esforço vocal e do hiperfuncionamento da musculatura laríngea nos participantes desse grupo, sendo este um efeito positivo de maior proteção à saúde vocal no exercício docente. Pesquisas correlacionam a redução da F_0_ à diminuição da tensão fonatória e à fala mais relaxada após a realização de exercícios vocais.^13^ ^,^ ^14^ ^,^ ^17^

Os resultados deste estudo indicam o possível efeito protetor do aquecimento vocal, uma vez que, inversamente, a elevação da F_0_ estaria relacionada ao aumento do número de ciclos de vibração das pregas vocais, desencadeando maior atrito e fadiga vocal, desconforto laríngeo e esforço fonatório após atividade de sobrecarga vocal.^14^ ^,^ ^17^

A redução da F_0_ também foi previamente confirmada^10^ após a realização de exercícios posturais e de relaxamento cervical por professores que receberam treinamento em comparação ao grupo controle, que recebeu abordagem indireta, ratificando a redução da tensão vocal. O programa de AV incluiu exercícios posturais e de alongamento cervical e de ombros que podem ter contribuído para a redução de F_0_.

Na análise intragrupo de TR, observou-se o contrário do esperado. Ao invés do treino respiratório ter proporcionado maior controle expiratório na propulsão do ar, pois se infere maior condicionamento da musculatura respiratória expiratória, os participantes deste grupo aumentaram significativamente o valor médio da medida de perturbação e variabilidade da amplitude de vibração, ciclo a ciclo (*shimmer*), indicando aumento da instabilidade fonatória.^2^

Os indicadores da análise acústica computadorizada da voz, nos momentos pré e pós-intervenção, não diferiram significantemente entre os grupos AV e TR.

Esperava-se que o AV apresentasse maior efetividade na qualidade vocal, indicando sua superioridade em relação ao TR, sendo provável que o número de sujeitos não tenha sido o suficiente, induzindo ao erro tipo II (falso negativo).

A maioria dos professores do grupo AV referiu melhora geral da voz e maior facilidade para falar após o seguimento, sendo constatada diferença estatisticamente significante em relação ao grupo TR. Esses resultados estão em consonância a estudos experimentais^4^ ^,^ ^15^ que encontraram maiores benefícios (melhora geral da voz e melhora da disfonia) relatados por professores que realizaram abordagem direta de intervenção.

Os benefícios autorreferidos pelo grupo AV podem estar relacionados ao recrutamento direto da musculatura fonatória realizado nos exercícios vibratórios, fricativos e ressonantais no intuito de equilibrar a emissão e proporcionar maior projeção, resistência e flexibilidade para o uso da voz por longos períodos de tempo.^7^ ^,^ ^10^

Por outro lado, o fato de o TR atuar de forma indireta no aparelho fonador, i.e., por recrutamento da musculatura respiratória expiratória,^5^ ^,^ ^18^ pode ter contribuído para que os participantes deste grupo não tenham observado benefícios no seu funcionamento vocal.

A amostragem utilizada neste estudo foi não probabilística e de conveniência, dificultando a generalização dos resultados para a população de professores. No entanto, a randomização suscita a garantia de que variáveis confundidoras tenham a mesma chance de alocação em qualquer um dos grupos, tornando-os comparáveis e minimizando os vieses de seleção e de confundimento.

A análise vocal não possui um desfecho único, robusto o suficiente para indicar mudanças na voz, sendo utilizados vários desfechos, os quais podem induzir ao erro tipo I (falso positivo).

Na última semana de seguimento da intervenção, os professores encontravam-se em menor demanda vocal devido à ausência de funcionários de limpeza da escola. Entretanto, ressalta-se que os sujeitos dos dois grupos randomizados estavam sob a mesma condição.

O efeito do trabalhador sadio, comum em estudos ocupacionais, pode ser a justificativa para a baixa ocorrência de professores com alterações vocais moderadas ou intensas autorreferidas antes das intervenções. Esses sujeitos poderiam estar afastados, readaptados ou terem abandonado a atividade devido à sintomatologia vocal, sendo subestimada a real frequência do fenômeno estudado.^1^ ^,^ ^16^

Os participantes do grupo AV podem ter referido maiores benefícios do aquecimento vocal no questionário pós-intervenção, influenciados psicologicamente pela maior expectativa de melhora da qualidade vocal com esta abordagem fonoaudiológica. Entretanto, a análise acústica dos parâmetros objetivos da voz supera esse provável viés.

Embora tenha sido minoria, os professores que relataram pouca adesão à abordagem proposta podem ter contribuído negativamente para a análise comparativa dos efeitos das intervenções. Sabe-se que a adesão à terapia de voz é um desafio e depende das expectativas de resultado, interesse e confiança em melhorar o comportamento vocal, podendo interferir no prognóstico e evolução do tratamento.^11^

Conclui-se que os grupos de intervenção não diferiram significantemente quanto aos indicadores de autoavaliação e acústicos da voz estudados, não sendo possível identificar uma das abordagens como mais efetiva e de maior proteção à saúde vocal dos professores. No entanto, isoladamente, tanto o aquecimento vocal quanto o treino respiratório produziram redução no IDV-10, autorreferido pelos professores. Os sujeitos do grupo AV ainda apresentaram redução de F_0_ e referiram maior benefício da intervenção para a melhora geral da voz e facilidade para falar após o seguimento.

Para a confirmação de nossos resultados, recomenda-se a realização de outros estudos experimentais sobre o tema, com maior número de sujeitos, envolvendo docentes de diferentes escolas.

## Footnotes

a Ministério da Saúde, Secretaria de Vigilância em Saúde, Departamento de Saúde Ambiental e Saúde do Trabalhador. Distúrbio de voz relacionado ao trabalho. Brasília (DF); 2011 [citado 2015 abr 26]. (Série A. Normas e Manuais Técnicos). Disponível em: http://aborlccf.org.br/imagebank/Protocolo_DVRT.pdf
